# mHealth-Based Diabetes Prevention Program for Chinese Mothers With Abdominal Obesity: Randomized Controlled Trial

**DOI:** 10.2196/47837

**Published:** 2025-01-24

**Authors:** Qinyuan Huang, Qinyi Zhong, Yanjing Zeng, Yimeng Li, James Wiley, Man Ping Wang, Jyu-Lin Chen, Jia Guo

**Affiliations:** 1 Xiangya School of Nursing Central South University Changsha China; 2 Department of nursing The 921st Hospital of Chinese People's Liberation Army (Second Affiliated Hospital of Hunan Normal University) Changsha China; 3 Manchester Centre for Health Psychology University of Manchester Manchester United Kingdom; 4 University of California, San Francisco San Francisco, CA United States; 5 School of Nursing University of Hong Kong Hong Kong China (Hong Kong); 6 School of Nursing University of California, San Francisco San Francisco, CA United States

**Keywords:** type 2 diabetes, mHealth, obesity, prevention, mothers

## Abstract

**Background:**

Among people with abdominal obesity, women are more likely to develop diabetes than men. Mobile health (mHealth)–based technologies provide the flexibility and resource-saving opportunities to improve lifestyles in an individualized way. However, mHealth-based diabetes prevention programs tailored for busy mothers with abdominal obesity have not been reported yet.

**Objective:**

The aim of this study is to evaluate the feasibility and acceptability of an mHealth-based diabetes prevention program and its preliminary efficacy in reducing weight-related variables, behavioral variables, psychological variables, and diabetes risk among Chinese mothers with abdominal obesity over 6 months.

**Methods:**

A randomized controlled trial was conducted at health management centers in 2 tertiary hospitals in Changsha, China. The mHealth group (n=40) received 12 weekly web-based lifestyle modification modules for diabetes prevention, 6 biweekly individualized health education messages based on their goal settings, and a Fitbit tracker. The control group (n=40) received 12 weekly web-based general health education modules, 6 biweekly general health education messages, and a Fitbit tracker. Data were collected at baseline, 3 months, and 6 months on the feasibility and acceptability outcomes, weight-related variables (waist circumference and BMI), diabetes risk scores, glycemic levels, behavioral variables (daily step count, active minutes, fruit and vegetable intake, calorie consumption, and sleep duration), and psychological variables (self-efficacy and social support for physical activity and diet, perceived stress, and quality of life). Generalized estimating equations were used for data analysis.

**Results:**

Approximately 85% (68/80) of the participants completed 6 months of follow-up assessments. Regarding the feasibility and acceptance of the program in the mHealth group, the average number of modules reviewed was 7.9 out of 12, and the satisfaction score was 4.37 out of 5. Significant improvements at 6 months between the intervention and control groups were found in waist circumference (β=–2.24, 95% CI –4.12 to –0.36; *P*=.02), modifiable diabetes risk scores (β=–2.5, 95% CI –4.57 to –0.44; *P*=.02), daily steps (β=1.67, 95% CI 0.06-3.29; *P*=.04), self-efficacy for physical activity (β=1.93, 95% CI 0.44-3.43; *P*=.01), social support for physical activity (β=2.27, 95% CI 0.80-3.74; *P*=.002), and physical health satisfaction (β=0.82, 95% CI 0.08-1.55; *P*=.03). No differences were found in BMI, total diabetes risk score, daily active minutes, daily intake of fruits and vegetables, sleep duration, daily calorie consumption, self-efficacy, and social support for diet (*P*>.05).

**Conclusions:**

This study addresses the potential role of tailored lifestyle interventions based on mHealth technology by offering tailored web-based health modules and health information in managing diabetes risk among mothers with abdominal obesity. The mHealth diabetes prevention program provides a flexible, customized, and resource-saving model for busy mothers. Future research could further explore the efficacy improvement on dietary behaviors to better serve the health care needs of this population.

**Trial Registration:**

Chinese Clinical Trial Registry ChiCTR2400090554; https://www.chictr.org.cn/showproj.html?proj=226411

## Introduction

Diabetes is the leading cause of death [1], accounting for 12.2% of all deaths in 2021 [2]. Approximately 537 million people have reported diabetes worldwide. There are complicated and various risk factors for developing diabetes or prediabetes, including aging, family history, unhealthy lifestyle, gut metagenome, and being overweight or obese [3,4]. By far, the strongest risk factor is being obese [5]. Moreover, compared to peripheral obesity, abdominal obesity is more closely associated with insulin resistance and metabolic disorders because the metabolic activity of visceral fat located in the abdomen is much higher than that of subcutaneous fat [6]. The waist circumference (WC) reflects the degree of abdominal fat accumulation in individuals [7]. Therefore, the reduction of WC plays a role in preventing and delaying the onset and progression of diabetes.

China has the largest diabetic population in the world [8]. According to a recent national study in 2020, a substantial number of new-onset type 2 diabetes mellitus cases in Chinese adults can be attributed to abdominal obesity, accounting for 50.4% in women and 30.3% in men [9]. Mothers rarely had free time to participate in face-to-face health promotion programs [10], albeit abdominal obesity is highly prevalent among middle-aged mothers with a normal BMI [11,12]. How to prevent diabetes among abdominally obese people has become an increasingly critical question in China, especially among mothers.

A healthy lifestyle, including low-calorie diets, high levels of physical activity, and self-management to lose weight or WC, benefits high-risk groups with diabetes. It is an effective approach to diabetes prevention [13]. For women with abdominal obesity, 2 lifestyle modification programs in the form of face-to-face diet and exercise coaching were effective in reducing their risk of developing diabetes [14,15]. However, interventions via face-to-face approaches require a large number of highly qualified health professionals to achieve the desired results [10]. The shortage of primary health care professionals and the strain on medical resources in low-income countries make it difficult to implement such interventions on a large scale for people at risk for diabetes [16]. Therefore, there is a great need to explore more flexible and resource-saving lifestyle modification interventions.

Mobile health (mHealth)–based and tailored health promotion programs via the WeChat platform have been widely used in diabetes and obesity management and have achieved satisfactory results [17,18]. However, these health promotion programs for lifestyle change are mostly used for people who have been diagnosed with diabetes [19]. There is little evidence of using mHealth technology for preventing diabetes [20]. For people with high risk but not diagnosed with diabetes, less adherence to lifestyle change compared to people with diabetes has been reported because they usually have lower awareness and lesser motivation to make changes [21]. In other words, whether lifestyle modification interventions via mHealth technology could work among high-risk people for diabetes, especially among busy mothers who have abdominal obesity, is still an open question.

The goal of this study was to promote changes in modifiable diabetes risk factors, thereby improving metabolic levels and reducing the risk of diabetes. The primary objective was to evaluate the feasibility and the acceptability of an mHealth-based diabetes prevention program and its preliminary efficacy in reducing weight-related variables, that is, WC and BMI, and diabetes risk (type 2 diabetes risk score) among Chinese mothers with abdominal obesity in 6 months. The secondary objective was to assess the preliminary efficacy of the intervention on glycated hemoglobin (HbA_1c_) levels, behavioral variables (daily steps and daily moderate to vigorous activity time), and psychological variables (self-efficacy and social support for physical activity and diet, perceived stress, and quality of life) at 6 months.

## Methods

### Ethics Approval

This study received approval from the ethics review committee of the Xiangya School of Nursing, Central South University (batch 2019002). This study was retrospectively registered with the Chinese Clinical Trial Registry (ChiCTR2400090554) on October 24, 2024.

### Study Design and Participants

This study utilizes a randomized controlled trial design. The inclusion criteria for the participants were as follows: (1) women as defined by biological sex, aged 18 years and older; (2) having access to a smartphone (Android and iOS phones compatible with Fitbit) and being willing to wear a Fitbit sports tracker for the entire study period; (3) willing to share their behavioral data collected via Fitbit with researchers in this study; (4) WC>80 cm and BMI>24.5; (5) having at least 1 child in the age range of 1-12 years; (6) not reached menopause; (7) able to read Chinese and speak Mandarin; and (8) planning to live locally for at least 8 months. The exclusion criteria were as follows: (1) being pregnant, (2) having given birth within 12 months prior to the enrollment date, (3) having an acute or life-threatening illness (eg, kidney failure), (4) having a condition that requires dietary and activity control (eg, diabetes, hypertension, hyperthyroidism), (5) having irregular periods for 6 months, and (6) having plans to become pregnant within 1 year.

### Sample Size

Monte Carlo simulations were conducted, aiming to estimate the power for effect sizes to be tested with multilevel regression models. Using G*power 3.1 software, sample size estimation was conducted based on the effect size of HbA_1c_. The effect size of HbA_1c_ was calculated according to relevant literature as 0.83 [22], using a 2-tailed test with a significance level set at .05. To ensure a power of 90%, the total sample size required was determined to be 64. Accounting for a 20% dropout rate, the total sample size needed for this study was 80 individuals, with 40 participants in each group.

### Recruitment

Participants were recruited from Health Management Centers for Adults and Children of 2 tertiary hospitals in Changsha, a provincial capital city of Hunan Province in China via posters and web-based recruitment. Two research assistants put up posters at the health management centers. The web-based recruitment was promoted in the WeChat Moments through Eqxiu. Eqxiu is an all-in-one creative design and marketing platform that uses design tools such as posters, long pages, and videos to produce marketing content for users. Interested mothers could contact the research assistants at the research sites. Research assistants measured the WC, height, and weight onsite to ensure they met inclusion and exclusion criteria.

If interested participants met the eligibility criteria, the researchers would provide them with a description of the purpose, content, process, risks, benefits, and the right to withdraw. Written informed consent was obtained from the participants in this study. All research-related information, including basic demographic data of the research participants and the original data recorded in this study, strictly adhered to the principles of research confidentiality. Individuals participating in this study received a designated compensation for the time and effort they expended in support of this research following the completion of each data collection session.

### Randomization

Randomization was performed using a random sequence generated by SPSS software (v. 25.0; SPSS Inc) in this study. The mHealth group was designated as number 1 beforehand and the control group as number 2. According to the time order of entry into the study, the researchers used SPSS software to generate a corresponding random number for each study participant and then coded half of the random numbers as 1 and the other half as 2. The study participants were entered into 2 groups according to this code. After randomization, the intervention and control groups included 40 participants, respectively. Neither the study participants nor the medical staff involved in recruitment were informed of the randomization allocation results. All research assistants involved in this study possessed extensive clinical and research experience. Prior to the commencement of the program, they underwent specialized training provided by the program lead. Notably, the research assistants responsible for the interventions in the mHealth group and the control group were different, with each receiving separate training to avoid potential contamination.

### mHealth Group Intervention

The mHealth-based lifestyle intervention was developed based on the social cognitive theory. The intervention consisted of 3 components: (1) recording participants’ exercise, sleep, and diet data via Fitbit during the whole study period (6 months); (2) accessing the 12 weekly lifestyle modification modules delivered on WeChat; and (3) receiving personalized biweekly messages after the modules, informed by Fitbit data, which were sent via WeChat for 3 months.

#### Fitbit Tracker (6 months)

Participants received personalized, one-on-one instruction from research assistants on using the Fitbit tracker and its mobile app prior to wearing the device. This instruction covered setting behavioral goals, learning to monitor and record behavior-related data systematically, interpreting displayed behavior values, and synchronizing/uploading data. Research assistants sent reminders via WeChat to participants who did not synchronize their data for over a week to improve compliance. Throughout the intervention, participants wore Fitbit trackers to record steps, moderate to vigorous activity time, and sleep. They could also manually enter dietary intake, automatically converted into calories. Fitbit, available as a mobile app for Android and iOS, facilitated automatic data syncing via Bluetooth. Individual sync data were accessible to researchers on the Fitbit website. Fitbit Alta HR trackers were obtained directly from the official Fitbit website using project funds. No additional payments were made to Fitbit, and participants incurred no usage fees. Chargers were provided with the trackers, and participants received both for free after the intervention.

WeChat is a Chinese instant messaging, social media, and mobile payment app with over 1 billion monthly active users. The rationale for disseminating educational modules to participants via WeChat stems from its ubiquitous presence as an everyday app for nearly all Chinese individuals owning a smartphone in China. Leveraging this platform ensures that each participant promptly receives our educational modules. The widespread use of WeChat in the daily lives of the Chinese population facilitates efficient and timely communication of educational content, maximizing the accessibility and reach of our intervention.

#### Weekly Educational Modules Posted on WeChat (12 Weeks)

**Figure 1:**
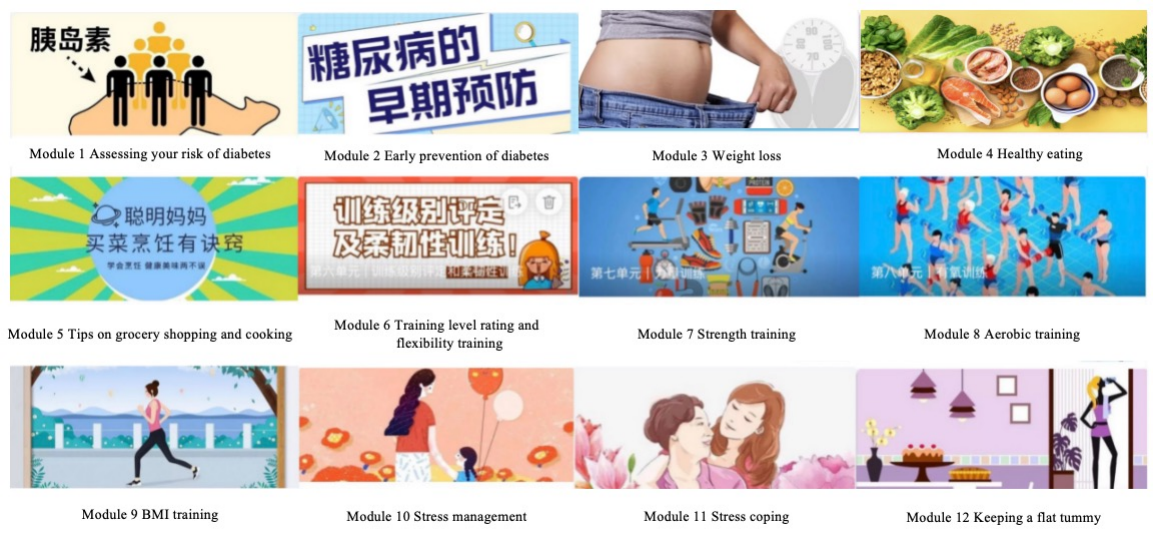
WeChat weekly educational modules for the mobile health group.

The researcher sent out 1 educational module per week via WeChat for 12 weeks. The educational modules consist of the following 5 categories: (1) introduction to diabetes and obesity (modules 1-3), (2) healthy diet (modules 4-5), (3) various exercise patterns (modules 6-9), (4) stress (modules 10-11), and (5) weight maintenance strategies (module 12). The selection of topics and content for the 12 educational modules was based on the etiology and factors contributing to diabetes [23], evidence from the American Diabetes Association, and consensus decisions reached by our expert panel. Figure 1 shows the details of the weekly educational modules. Each exercise module consists of 3 videos of varying difficulty levels: low, moderate, and high. Participants can select the appropriate level based on their individual fitness capabilities. All modules can be viewed repeatedly. Research assistants remind participants to review modules promptly with messages and phone calls. A point system, rewarding 10 points per module, motivated ongoing participation, with a small gift at 120 points.

#### Tailored WeChat Messages Based on Individual Behavioral Patterns and Preferences (6 Messages in Total)

Research assistants assessed exercise and dietary patterns for tailored messages based on the recordings. After completing the 12 weekly web-based educational modules on WeChat, the research team analyzed the Fitbit data of participants in the mHealth group in order to send tailored messages aligned to the participant’s behavioral patterns based on their exercise and diet data for the last 2 weeks. According to the participants’ situations of goal completion, individual behavioral patterns were categorized into 3 levels: below target, meeting target, and exceeding target. Exercise steps up to 0%-70% of the target steps were identified as below target, 71%-99% were identified as meeting target, and 100% and above were identified as exceeding target. Dietary targets were set according to the dietary guidelines: total daily intake of vegetables and fruits was set at 5 standard servings (approximately 500 g, 100 g raw weight per standard serving), with 1-2 servings identified as below target, 3-4 servings identified as meeting target, and 5 servings and above identified as exceeding target. Tailored biweekly WeChat messages consisted of 3 components: assessment of current progress, transition words, and recommendations. For instance, “Congratulations, Ms Wang! You’ve reached your exercise goal, moving closer to a healthier lifestyle. Find more dietary advice on module 4 ‘Healthy Eating.’ Keep up the good work!”

### Control Group Interventions

**Figure 2:**
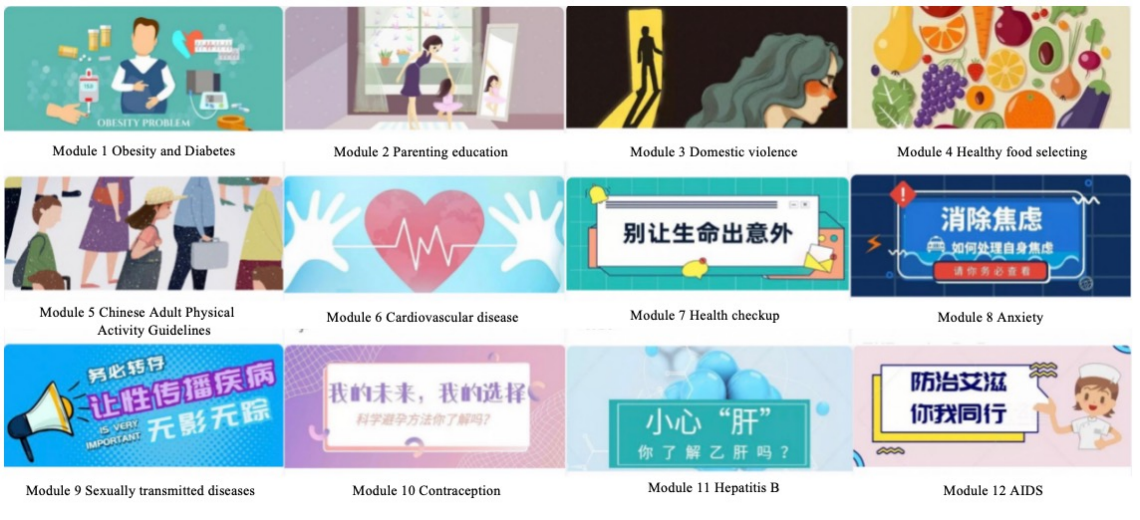
WeChat weekly educational modules for the control group.

The control group interventions consisted of 3 main components: Fitbit trackers identical to those used by the mHealth group, 12 educational modules addressing general health information delivered on WeChat, and health-improvement messages. The 12 modules, which did not focus on lifestyle modification, covered a variety of topics: (1) obesity and diabetes, (2) parenting education, (3) domestic violence, (4) sourcing food, (5) Chinese Adult Physical Activity Guidelines, (6) cardiovascular disease, (7) health checkups, (8) anxiety, (9) sexually transmitted diseases, (10) contraception, (11) hepatitis B, and (12) AIDS. Figure 2 shows the samples of the weekly educational modules for the control group. The supervision pattern for module completion in the control group mirrored that of the mHealth group. Additionally, nontailored informational tips were delivered to the control group, aligning with the educational content of the modules. For instance, participants received messages such as “AIDS, a class B legal infectious disease in China, lacks effective cure drugs. Click to review the tenth module on WeChat.”

### Data Collection

At baseline, 3 months, and 6 months, research assistants who were unaware of group allocation contacted the participants via phone or WeChat for data collection, including questionnaires and physical examinations. The research assistants responsible for contacting participants were distinct from those who implemented the interventions and were blinded to group assignments. Participants completed questionnaires in a designated hospital room, with researchers available for assistance. Physical examinations, conducted by trained nurses, included height (measured at baseline), weight, and WC, with data averaged from 3 measurements. Fasting blood samples for HbA_1c_ were collected by hospital nurses at baseline and 6 months. Participants received travel and meal allowances as appreciation for their participation. To avoid contamination, data collection for both groups occurred on separate dates at the study sites.

### Outcomes Measures

#### Feasibility and Acceptability

The feasibility was evaluated by calculating the number of module views by participants, which is automatically recorded by the WeChat platform. The acceptability outcome measure is a satisfaction question concerning each module. Satisfaction questions asked participants whether they thought they had learnt something after watching the content of a module, with scores ranging from 1 to 5—a higher score indicating a higher level of satisfaction.

#### Demographics

General sociodemographic information was collected at baseline, consisting of 26 questions on the study population’s demographic and sociological information, including maternity history, lifestyle habits, and family history of diabetes and other chronic diseases.

#### Measurement of Weight-Related Variables

WC is the circumference of the horizontal line between the upper edge of the iliac crest and the lowest point of the rib cage—generally the horizontal circumference of the center of the umbilicus. A trained nurse instructed the participant to breathe regularly and read the value of the soft ruler (accuracy of 0.1 cm) at the end of exhalation. The value was recorded as the average of the measurements. BMI is calculated by dividing body weight (kg) by the square of height (m). Height is measured using the same calibrated height gauge (0.5 cm accuracy) and weight scale (0.1 kg accuracy).

#### Measurement of Diabetes Risk Score

The Chinese Diabetes Risk Questionnaire (CHINARISK) as the diabetes risk assessment tool consists of 14 modifiable and unmodifiable items covering age, gender, ethnicity, residence, education level, WC, BMI, daily exercise, daily intake of fruits and vegetables, history of hypertension, treatment history of taking medication for hypertension, history of giving birth to a large child, blood glucose status, and family history of diabetes. Among these, the 4 modifiable risk factors were WC, BMI, daily exercise, and daily intake of fruits and vegetables. The scores of the 4 entries were summed to calculate the CHINARISK modifiable scores. The modifiable score implies that by modifying lifestyle and achieving weight loss, it is possible to lower the scores in CHINARISK, thereby contributing to a reduction in the risk of diabetes. The possible total score range of CHINARISK is 0-82 (including the modifiable and unmodifiable scores). A total score above 30 is considered a high risk of developing diabetes in the future and requires prompt intervention and further diagnosis. The retest reliability of CHINARISK was 0.988, with a positive predictive value of 57%, a negative predictive value of 78%, and sensitivity of 73% [24].

#### Measurement of Metabolic Variables

HbA_1c_ reflects average blood glucose levels over 2-3 months, with a normal range of 4%-5.6% [25]. Fasting blood specimens were sent to Changsha Goldfield Medical Testing Center within 2 hours for standardized testing, thereby reducing methodological errors across sites. Standardized HbA_1c_ reports were issued by the Goldfield Medical Testing Center.

#### Measurement of Behavioral Variables

All behavioral variables in this study, including step count, moderate to vigorous activity time, sleep duration, daily fruit and vegetable intake, and total daily calorie intake/burn, were recorded in real time by the Fitbit tracker. It accurately measures step counts through a built-in gyroscope and acceleration sensors and tracks moderate to vigorous activity time by using the accelerometer. Sleep duration is determined by the frequency and amplitude of tossing and turning. The Fitbit tracker estimates daily calorie intake/consumption from exercise time, intensity, sleep duration, BMI, etc. It has shown good reliability and validity in various large lifestyle intervention studies, with intraclass correlation coefficients ranging from 0.71 to 1.00 [26]. In this study, daily steps and daily moderate to vigorous activity time were averaged over the last week at baseline, 3 months, and 6 months.

#### Measurement of Diet and Exercise-Related Psychological Variables

The Dietary and Physical Self-Efficacy Scale and Social Support Scale are used to assess participants’ self-efficacy and the level of social support provided by family and friends in relation to dietary and exercise behaviors [27,28]. The Self-Efficacy Scale and the Social Support Scale has 4 and 6 entries, respectively. These 4 scales are scored on a 4-point scale, with higher scores indicating higher levels of individual self-efficacy and social support for eating and exercise. The Cronbach coefficients for the Chinese version of the Dietary Self-Efficacy Scale and the Physical Activity Self-Efficacy Scale ranged from 0.93 to 0.95 and 0.87 to 0.97, respectively. The Cronbach coefficients of the Chinese version of the Dietary Social Support Scale and the Physical Activity Social Support Scale ranged from 0.86 to 0.89 and 0.78 to 0.87, respectively. Perceived Stress Scale-14 items (PSS-14) is used to assess the amount of stress perceived by the participant over the past month [29]. The scores for each item are summed to give a total score on the PSS-14, which ranges from 0 to 56. The higher the score, the higher the perceived stress. The Cronbach coefficient of the Chinese version of the PSS-14 scale was 0.78.

#### Measurement of Quality of Life

The World Health Organization Quality of Life Scale-Brief Version is used to quantify participants’ self-assessment of their own quality of life and general health status [30]. It contains a total of 26 items and addresses 4 quality of life domains: physical health (7 items), psychological health (6 items), social relationships (3 items), and environment (8 items). The items are scored on a 5-point Likert scale. Higher scores in a domain indicate higher self-rated quality of life in that domain. Domain scores range from 4 to 20, with a Cronbach coefficient of 0.88 for the Chinese version of World Health Organization Quality of Life Scale-Brief Version.

### Statistical Methods

Raw data were entered in pairs using EpiData 3.1 software [31] to verify accuracy. Data were analyzed using SPSS 25.0 software [32] with a test level of α taken as .05. Statistical description methods were proposed to describe the demographic and sociological data and all scale data for both study groups. One-way analysis of variance (for measurement information), least significance difference tests (2-way comparisons between factors), and *χ*^2^ tests (for counting information) were performed to compare the differences in demographic information and primary and secondary variables between the 2 groups of enrolled and study participants who did not complete the follow-up assessments. Intention-to-treat analysis was used, with all participants (N=80) analyzed based on their original group assignments, including those excluded or lost. To compare outcome indicators (including psychological, behavioral, weight-related, and diabetes risk–related variables) between the 2 groups over 6 months, we established a 3 (time: baseline, 3 months, and 6 months) * 2 (group: intervention and control) generalized estimating equation (GEE) model by using general sociodemographic information and disease risk–related data as covariates to control for the confounding factors. Additionally, we developed a 2 (time: baseline and 6 months) * 2 (group: intervention and control) GEE model to evaluate the time trend of HbA_1c_ levels and compare the differences in HbA_1c_ between the mHealth group and the control group.

The GEE model offers key advantages over traditional repeated measures analysis. It accounts for within-subject correlations in longitudinal data, provides reliable estimates even when the correlation structure is incorrectly specified, and focuses on population-average effects. GEE can also handle missing data by assuming it occurs completely at random, allowing for the inclusion of incomplete datasets. Additionally, by incorporating covariates, GEE controls for confounding factors, making it a robust choice for analyzing repeated measurements over time.

## Results

### Baseline Sociodemographic and Clinical Characteristics

**Figure 3:**
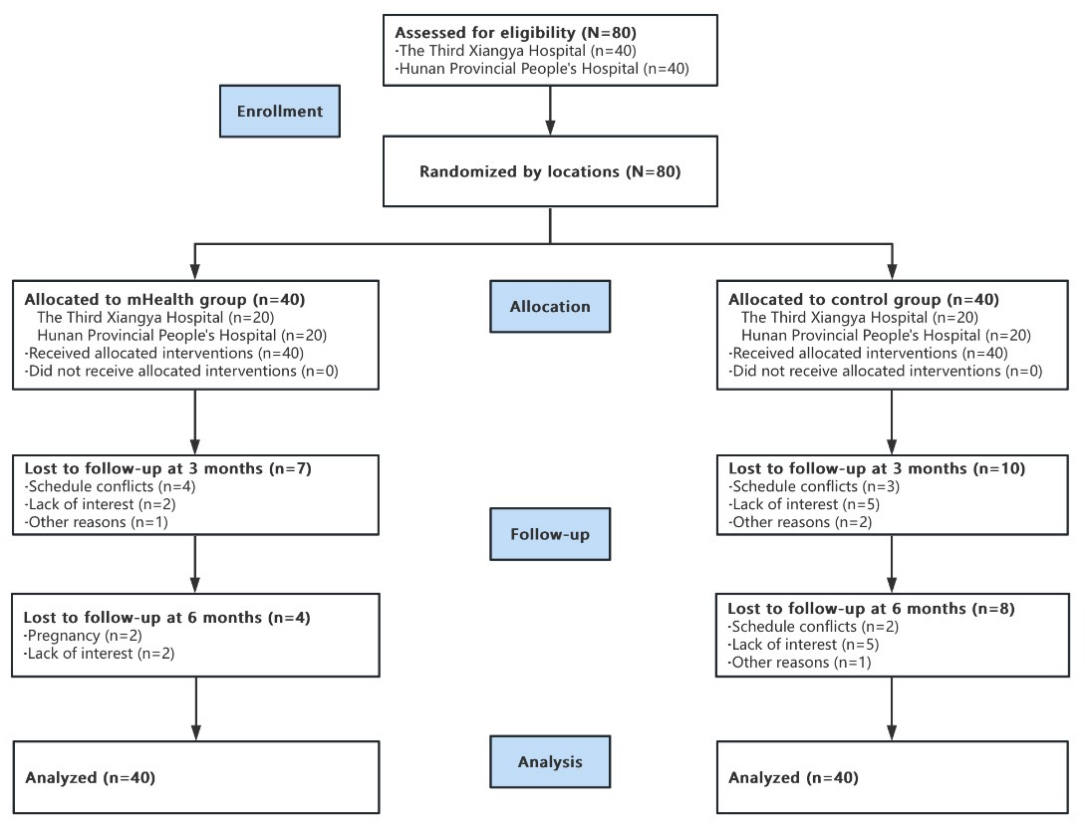
Study design and participant flow.

A total of 80 participants were enrolled and randomly assigned to the mHealth group (n=40) and the control group (n=40). Figure 3 lists the details of inclusion and loss to follow-up. The mean age of the participants was 34.86 (SD 4.36; range 26-45) years. About 79% (63/80) of the participants had a university or higher level of education. The mean WC and BMI of the sample were 92.61 (SD 9.58) cm and 28.12 (SD 3.93) kg/m^2^, respectively. The mean type 2 diabetes risk score of the sample was 21.50 (SD 9.66). There were no statistically significant differences in baseline sociodemographic and clinical characteristics between the mHealth and control groups. There were no significant differences in the demographic and clinical characteristics between participants who completed the follow-ups and those who missed the follow-ups (*P*>.05; Table 1).

**Table 1:** Comparison of the baseline sociodemographic and clinical characteristics between the control and mobile health groups.

Variables			Total (N=80)		Mobile health group (n=40)		Control group (n=40)		*χ*^2^/*t* test *(df)*			*P* value	
**Age (years), mean (SD)**			34.86 (4.36)		34.56 (4.01)		35.19 (4.77)		0.63 (77)^a^			.53	
**Education, n (%)**										1.9 (1)^b^			.17
	Senior high school and below	17 (21)		6 (15)		11 (28)							
	College and above	63 (79)		34 (85)		29 (72)							
**Marital status, n (%)**										0.2 (1)^b^			.64
	Married	75 (94)		37 (92)		38 (95)							
	Divorced	5 (6)		3 (8)		2 (5)							
**Occupation, n (%)**										0.49 (1)^b^			.49
	Employed	51 (64)		27 (67)		24 (60)							
	Unemployed	29 (36)		13 (33)		16 (40)							
**Youngest child age (years), mean (SD)**			5.99 (2.98)		5.78 (3.01)		6.22 (2.98)		0.65 (75)^a^			.52	
**BMI (kg/m^2^), mean (SD)**			28.12 (3.93)		28.19 (4.12)		28.05 (3.76)		–0.15 (76)^a^			.88	
**Waist circumference (cm), mean (SD)**			92.61 (9.58)		93.09 (10.45)		92.07 (8.63)		–0.47 (76)^a^			.64	
**Hemoglobin A_1c_, mean (SD)**			5.74 (0.74)		5.83 (0.93)		5.65 (0.45)		–1.06 (74)^a^			.29	
**Five servings of fruits and vegetables daily intake, n (%)**										1.4 (1)^b^			.25
	Yes	29 (36)		12 (30)		17 (43)							
	No	51 (64)		28 (70)		23 (57)							
**30 minutes of daily exercise, n (%)**										0.4 (1)^b^			.50
	Yes	35 (44)		19 (48)		16 (40)							
	No	45 (56)		21 (52)		24 (60)							
**Type 2 diabetes risk score, mean (SD)**			21.50 (9.66)		21.46 (9.05)		21.54 (10.42)		0.04 (76)^a^			.97	

^a^*t* test values (2-sided).

^b^Chi-square values.

### Feasibility and Acceptability

The total number of WeChat module views for the mHealth and control groups were 697 and 189, respectively, and the top 3 view sessions in the mHealth group were modules 1, 6, and 3 with 89, 80, and 77 views, respectively. All modules can be viewed repeatedly but will also be counted repeatedly. The mean score of the satisfaction questionnaire about weekly educational modules posted on WeChat was 4.37 out of 5 for the mHealth group and 3.71 for the control group.

### Changes in the Primary and Secondary Outcomes Over Time Between mHealth and Control Groups

**Figure 4:**
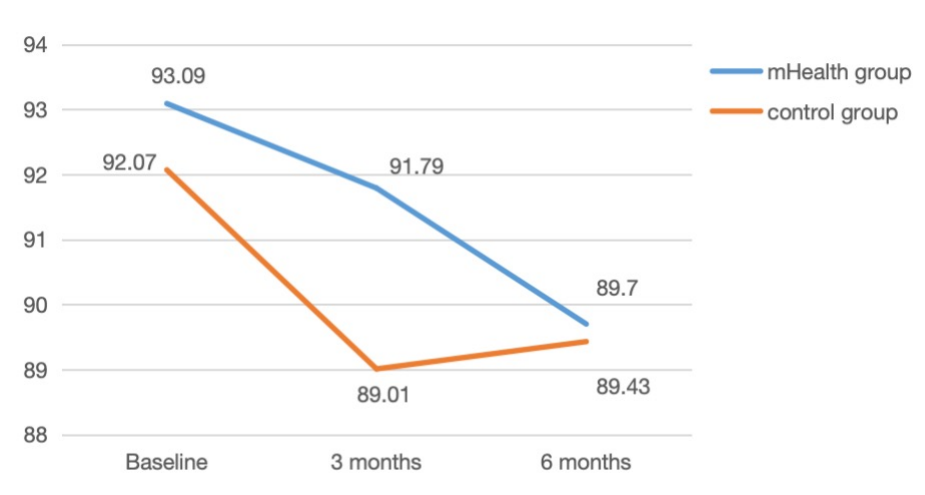
Changes in the waist circumference between the mobile health and control groups over 6 months. mHealth: mobile health.

**Figure 5:**
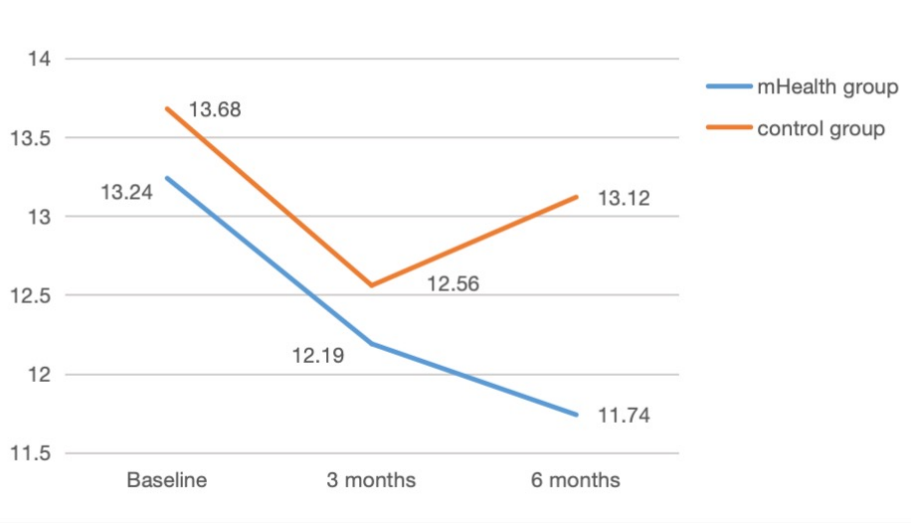
Changes in the modifiable type 2 diabetes mellitus risk scores between the mobile health and control groups over 6 months. mHealth: mobile health.

With a reduction of 3.39 cm and 1.50 points in the mHealth group versus 2.37 cm and 0.56 points in the control group at the 6-month follow-up (Figures 4 and 5; Table 2), respectively, we evaluated the effectiveness of the intervention and accounted for missing data through an intention-to-treat analysis.

The intention-to-treat analysis of the adjusted GEE model revealed a significant group-by-time interaction effect on WC and modifiable type 2 diabetes risk scores (Table 3). We observed a gradual decrease in the primary outcome measures due to the group-by-time interaction effect. No significant effect on BMI was found (Table 3). For secondary outcomes, there was a significant group-by-time interaction effect for the mHealth group on daily steps, self-efficacy, social support for physical activity, and the physiological domain of quality of life (Table 3). Specifically, the daily steps and daily moderate to vigorous activity time in the mHealth group were significantly higher than those in the control group at baseline, 3 months, and 6 months. Although the daily moderate to vigorous activity time in the mHealth group was consistently higher than that in the control group, there was no significant group-by-time interaction effect for this variable (Table 2 and Table 3).

**Table 2:** Comparison of the primary and secondary outcomes between the mobile health group and control group (N=80).

Variables		Mobile health group			Control group			
		Baseline (n=40)	3 months (n=33)	6 months (n=36)	Baseline (n=40)	3 months (n=30)	6 months (n=32)	
**Primary outcome, mean (SD)**								
	Waist circumference	93.09 (10.46)	91.79 (11.22)	89.70 (10.13)	92.07 (8.63)	89.01 (9.13)		89.70 (9.45)
	BMI	28.19 (4.12)	27.82 (6.79)	28.42 (4.49)	28.06 (3.77)	28.26 (3.70)		28.23 (3.51)
	Type 2 diabetes risk scores	21.46 (9.05)	21.09 (10.35)	21.26 (11.58)	21.54 (10.43)	20.88 (9.94)		21.88 (11.31)
	Modifiable type 2 diabetes risk scores	13.24 (4.43)	12.19 (5.11)	11.74 (5.75)	13.68 (5.13)	12.56 (5.86)		13.12 (5.64)
**Secondary outcomes, mean (SD)**								
	Hemoglobin A_1c_	5.84 (0.93)	N/A^a^	5.78 (1.07)	5.65 (0.46)	N/A		5.85 (1.61)
	Daily steps	10,662.11 (4542.38)	10,793.70 (4825.32)	8967.02 (6431.57)	8441.45 (3666.66)	8657.78 (4915.79)		4934.83 (3692.10)
	Daily moderate to vigorous activity time	40.06 (40.69)	42.09 (46.51)	31.82 (46.15)	27.49 (19.41)	15.25 (19.54)		5.63 (15.60)
	Self-efficacy for diet	6.73 (2.86)	6.91 (2.81)	6.32 (2.77)	6.32 (4.11)	6.70 (3.14)		6.88 (3.44)
	Self-efficacy for physical activity	6.98 (3.42)	6.31 (2.81)	6.47 (2.79)	7.43 (3.73)	6.06 (3.24)		6.27 (3.25)
	Social support for diet	5.00 (1.52)	5.66 (1.98)	4.97 (1.38)	5.24 (2.15)	5.31 (1.64)		4.76 (1.71)
	Social support for physical activity	11.51 (3.63)	11.91 (3.73)	11.50 (3.47)	12.68 (4.68)	11.53 (3.89)		11.61 (4.11)
	Perceived stress	23.68 (8.78)	25.31 (8.472)	23.53 (9.33)	22.57 (6.62)	22.66 (7.89)		23.91 (6.82)
	Physiological QoL^b^	14.04 (2.04)	13.20 (4.08)	14.06 (2.26)	14.10 (1.93)	13.95 (2.16)		14.06 (1.96)
	Psychological QoL	13.96 (2.35)	12.71 (4.23)	13.78 (2.69)	13.77 (1.98)	14.27 (1.94)		13.82 (1.80)
	Environment QoL	13.69 (2.21)	12.64 (4.02)	13.37 (2.00)	13.87 (1.94)	14.27 (1.84)		13.83 (1.80)
	Social relationships QoL	14.13 (3.01)	12.94 (4.07)	13.92 (2.25)	14.41 (2.06)	14.54 (2.18)		14.30 (2.57)

^a^N/A: not applicable. Hemoglobin A_1c_ was only measured twice in the program.

^b^Qol: quality of life.

**Table 3:** Generalized estimating equation analysis of the 6-month efficacy of the intervention on the primary outcomes and the secondary outcomes^a^.

Outcome				β estimate (SE; 95% CI)		*P* value
**Primary outcomes, mean (SD)**						
	**Waist circumference**					
		Group	4.90 (2.83; –0.65 to 10.46)		.08	
		Time	2.10 (1.61; –1.07 to 5.27)		.20	
		Group × time	–2.24 (0.95; –4.12 to –0.36)		.02	
	**BMI**					
		Group	1.23 (1.07; –0.88 to 3.34)		.26	
		Time	0.89 (0.50; –0.09 to 1.87)		.08	
		Group × time	–0.39 (0.26; –0.92 to 0.12)		.14	
	**Type 2 diabetes risk scores**					
		Group	6.82 (4.07; –1.16 to 14.80)		.09	
		Time	7.89 (4.91; –1.73 to 17.51)		.11	
		Group × time	–4.18 (2.57; –9.22 to 0.86)		.10	
	**Modifiable type 2 diabetes risk scores**					
		Group	2.74 (1.98; –1.14 to 6.63)		.17	
		Time	4.35 (2.04; 0.34 to 8.36)		.03	
		Group × time	–2.50 (1.05; –4.57 to –0.44)		.02	
**Secondary outcomes, mean (SD)**						
	**Hemoglobin A_1c_**					
		Group	0.32 (0.26; –0.18 to 0.84)		.21	
		Time	0.42 (0.46; –0.48 to 1.32)		.36	
		Group × time	–0.23 (0.23; –0.69 to 0.21)		.31	
	**Daily steps**					
		Group	0.56 (1.58; –2.54 to 3.67)		.72	
		Time	–4.82 (1.21; –7.19 to –2.45)		<.001	
		Group × time	1.67 (0.82; 0.06 to 3.29)		.04	
	**Daily moderate to vigorous activity time**					
		Group	0.39 (5.63; –10.64 to 11.44)		.94	
		Time	–9.11 (3.53; –16.03 to –2.18)		.01	
		Group × time	3.28 (2.25; –1.13 to 7.70)		.15	
	**Self-efficacy for diet**					
		Group	2.59 (1.99; –1.31 to 6.50)		.19	
		Time	3.33 (2.01; –0.62 to 7.28)		.10	
		Group × time	–1.52 (1.07; –3.63 to 0.58)		.16	
	**Self-efficacy for physical activity**					
		Group	–2.77 (1.65; –6.01 to 0.45)		.09	
		Time	–4.05 (1.27; –6.55 to –1.55)		.001	
		Group × time	1.93 (0.76; 0.44 to 3.43)		.01	
	**Social support for diet**					
		Group	–0.56 (0.96; –2.45 to 1.31)		.55	
		Time	–1.12 (1.03; –3.16 to 0.90)		.28	
		Group × time	0.51 (0.55; –0.56 to 1.59)		.35	
	**Social support for physical activity**					
		Group	–0.36 (1.45; –6.46 to –0.75)		.01	
		Time	–4.38 (1.32; –6.98 to –1.79)		.001	
		Group × time	2.27 (0.74; 0.80 to 3.74)		.002	
	**Perceived stress**					
		Group	2.02 (3.30; –4.45 to 8.51)		.54	
		Time	2.11 (3.37; –4.50 to 8.73)		.53	
		Group × time	–1.32 (1.88; –5.02 to 2.38)		.49	
	**Physiological quality of life**					
		Group	–1.04 (0.73; –2.48 to 0.39)		.15	
		Time	–1.59 (0.61; –2.80 to –0.38)		.01	
		Group × time	0.82 (0.37; 0.08 to 1.55)		.03	
	**Psychological quality of life**					
		Group	0.43 (0.81; –1.16 to 2.03)		.60	
		Time	0.89 (0.69; –0.46 to 2.25)		.20	
		Group × time	–0.45 (0.41; –1.26 to 0.36)		.28	
	**Environment quality of life**					
		Group	–0.02 (0.72; –1.44 to 1.39)		.97	
		Time	0.40 (0.30; –0.20 to 1.00)		.20	
		Group × time	–0.35 (0.24; –0.83 to 0.11)		.14	
	**Social relationships quality of life**					
		Group	–0.40 (0.81; –1.99 to 1.18)		.61	
		Time	0.07 (0.46; –0.83 to 0.98)		.88	
		Group × time	–0.04 (0.32; –0.67 to 0.58)		.89	

^a^When constructing the generalized estimating equation model, time was categorized into baseline, 3 months, and 6 months for all outcome indicators, except hemoglobin A_1c_. When hemoglobin A_1c_ was the dependent variable, time was categorized into baseline and 6 months. The participants were divided into 2 groups: intervention and control.

## Discussion

### Principal Findings

To the best of our knowledge, this is the first study to explore the use of mHealth technology for diabetes prevention in mothers with abdominal obesity in China. We demonstrated that a tailored mHealth-based diabetes prevention intervention is both feasible and resource-efficient, yielding significant positive effects. Specifically, our findings provide preliminary evidence that such interventions can reduce WC, increase daily step count, enhance exercise-related self-efficacy and social support, improve quality of life, and lower modifiable diabetes risk. The high level of compliance among participants suggests potential for implementation in primary health care centers.

The success of this program can be attributed to several key factors. First, the intervention was grounded in the social cognition theory [33], which posits that psychosocial influences on health behavior are shaped by 3 factors: goals, outcome expectancies, and self-efficacy. In this study, participants set realistic, personalized goals for diet and exercise, greatly enhancing the likelihood of achieving desired outcomes. In addition, Fitbit’s data visualization feature encouraged self-motivation and boosted participants’ self-efficacy [34]. Second, our study population consisted of mothers whose children were younger than 12 years, a stage when increased maternal involvement is often required in various aspects of life, including academics, physical health, and emotional well-being. At the same time, they were at a life stage marked by active professional pursuits. As a result, these mothers may have inadvertently neglected their own health due to the competing demands of family and work. However, the mHealth-based lifestyle management intervention overcame time and space constraints, enhancing adherence, particularly for busy mothers [35,36].

The mHealth-based, tailored diabetes prevention intervention effectively reduced WC in mothers with abdominal obesity, which was consistent with findings from a recent meta-analysis [37]. The intervention included components aimed at reducing body fat and WC, such as 12 web-based modules (4 on exercise and 3 on diet). The 15-minute exercise videos were designed for both individual use and joint participation with children. These short sessions made it easier for busy mothers to stay active daily. Additionally, wearing exercise trackers and receiving personalized health text messages further supported WC reduction. This approach highlights that small, consistent daily efforts play a key role in achieving long-term health improvement.

In this study, while the overall diabetes risk score did not show a significant reduction, it is noteworthy that a significant difference in diabetes risk scores emerged between the 2 groups when the 10 nonmodifiable risk factors from the CHINARISK diabetes risk scale were excluded. This left only 4 modifiable risk factors: WC, BMI, physical activity, and dietary habits. These findings suggest that tailored diabetes risk management interventions using mHealth technology can effectively target controllable risk factors such as WC, BMI, diet, and physical activity, ultimately helping to lower an individual’s future risk of developing diabetes.

Exercise self-efficacy, as an essential component of health self-efficacy, reflects an individual’s belief in their ability to resist temptations and maintain a healthy exercise routine [38]. This intervention led to significant improvements in exercise-related psychological variables, including exercise self-efficacy and exercise social support, which are likely to encourage women to engage in more physical activity. This aligns with findings from a systematic review of lifestyle interventions [39]. Our 12 web-based educational modules incorporated family-based exercise training, offering participants new formats that mobilize social resources and enhance support for exercise. However, there is limited national and international research on exercise-related social support for women with children at risk of diabetes. Therefore, the findings regarding social support in this study should be interpreted with caution.

This study shows that tailored mHealth-based diabetes risk management improved the quality of life in the physiological domain for abdominally obese mothers. Participants reported greater satisfaction with their sleep, increased energy levels for daily activities, and enhanced ability to perform everyday tasks. These findings align with weight management studies that link weight reduction to improved sleep quality [40]. Our intervention included exercise training, which encouraged behaviors such as increasing daily step counts. According to the pendulum effect [41], adequate physical activity during the day promotes deeper sleep at night, reduces sleep latency, and enhances overall sleep quality and satisfaction. This, in turn, leads to improved energy levels for daily activities, ultimately enhancing participants’ quality of life in the physiological domain.

The frequent module reviews and the high satisfaction ratings reflect the feasibility of our research. The traditional face-to-face diabetes risk management program is costly and time-consuming, posing challenges in low-income countries with a shortage of primary health care professionals and limited medical resources [16]. WeChat, a popular multifunctional mobile app, is China’s leading instant messaging tool and offers a wide range of connectivity features [42]. Utilizing WeChat as a web-based platform for health education has proven effective in promoting weight loss among Chinese obese women and even preventing breast cancer [43]. Our diabetes risk management program on WeChat leverages its flexible learning formats to engage participants and ensure high adherence to the intervention. Additionally, it requires less reliance on health care professionals, which helps conserve both human and economic resources—a significant advantage in the current context of low-income countries.

### Limitations

First, participants were recruited exclusively from Changsha, limiting the generalizability of the findings to mothers with abdominal obesity across China. Second, the study lasted only 6 months, which may be insufficient to fully understand the long-term development of diabetes, given its gradual onset. Third, there could be discrepancies between the participants’ actual eating behaviors and the diet data they manually entered in the Fitbit app. Fourth, the intervention and data collection mainly took place in autumn, a time when people tend to eat more [44], potentially contributing to the lack of significant changes in dietary variables in this study.

### Implications

Future trials should place importance on long-term follow-ups to confirm the sustained effectiveness of this risk management intervention. Using factorial design methodology can help identify which specific elements of the intervention directly influence changes in patient behavior. This study, delivered through the WeChat platform, uses flexible learning formats to actively engage participants and ensure high adherence to the intervention. Moreover, by reducing reliance on health care professionals, this approach provides cost-effective benefits, which are particularly valuable in resource-limited health care settings.

### Conclusion

This mHealth-based and tailored diabetes prevention model lays both the theoretical groundwork and practical strategies crucial for scaling up diabetes prevention and risk management initiatives in public health nursing practice. Although our study sheds light on the initial effects of mHealth-based interventions, it addresses the current scarcity of medical resources in China, offering a potential blueprint for tackling various chronic conditions.

## Appendix

Multimedia Appendix 1 CONSORT-EHEALTH (Consolidated Standards of Reporting Trials of Electronic and Mobile Health Applications and online Telehealth) checklist.

## Abbreviations

CHINARISK: Chinese Diabetes Risk Questionnaire
GEE: generalized estimating equation
HbA_1c_: glycated hemoglobin A_1c_
mHealth: mobile health
PSS-14: Perceived Stress Scale-14 items
WC: waist circumference

## Data Availability

The datasets generated and analyzed during this study are available from the corresponding author upon reasonable request.
